# A novel read methodology to evaluate the optimal dose of ^68^Ga-satoreotide trizoxetan as a PET imaging agent in patients with gastroenteropancreatic neuroendocrine tumours: a phase II clinical trial

**DOI:** 10.1186/s13550-021-00819-1

**Published:** 2021-09-06

**Authors:** Colin G. Miller, Henning Grønbæk, Irene Virgolini, Andreas Kjaer, Pierre Terve, Shadfar Bahri, Peter Iversen, Hanna Svirydenka, Thomas Rohban, Sandy McEwan

**Affiliations:** 1 The Bracken Group for Ipsen Bioscience, 12 Penns Trail, Newtown, PA 18940 USA; 2 Department of Hepatology and Gastroenterology, Aarhus University Hospital, Aarhus, Denmark; 3 Department of Nuclear Medicine, University of Innsbruck, Innsbruck, Austria; 4 Department of Clinical Physiology, Nuclear Medicine & PET and Cluster for Molecular Imaging, Department of Biomedical Sciences, Rigshospitalet, University of Copenhagen, Copenhagen, Denmark; 5 Keosys, Nantes, France; 6 Ahmanson Translational Theranostics Division, Department of Molecular and Medical Pharmacology, David Geffen School of Medicine, UCLA, Los Angeles, CA USA; 7 Department of Nuclear Medicine and PET Center, Aarhus University Hospital, Aarhus, Denmark; 8 Partner 4 Health for Ipsen Bioscience, Paris, France; 9 Ipsen Bioscience, Cambridge, MA USA

**Keywords:** ^68^Ga-satoreotide trizoxetan, Neuroendocrine tumours, Somatostatin receptor antagonist, Diagnostic imaging, Binary visual reading

## Abstract

**Background:**

^68^Ga-satoreotide trizoxetan is a novel somatostatin receptor antagonist exhibiting higher tumour-to-background ratios and sensitivity compared to ^68^Ga-DOTATOC. This randomised, 2 × 3 factorial, phase II study aimed to confirm the optimal peptide mass and radioactivity ranges for ^68^Ga-satoreotide trizoxetan, using binary visual reading. To that end, 24 patients with metastatic gastroenteropancreatic neuroendocrine tumours received 5–20 µg of ^68^Ga-satoreotide trizoxetan on day 1 of the study and 30–45 µg on day 16–22, with one of three gallium-68  radioactivity ranges (40–80, 100–140, or 160–200 MBq) per visit. Two ^68^Ga-satoreotide trizoxetan PET/CT scans were acquired from each patient post-injection, and were scored by experienced independent blinded readers using a binary system (0 for non-optimal image quality and 1 for optimal image quality). For each patient pair of ^68^Ga-satoreotide trizoxetan scans, one or both images could score 1.

**Results:**

Total image quality score for ^68^Ga-satoreotide trizoxetan PET scans was lower in the 40–80 MBq radioactivity range (56.3%) compared to 100–140 MBq (90.6%) and 160–200 MBq (81.3%). Both qualitative and semi-quantitative analysis showed that peptide mass (5–20 or 30–45 µg) did not influence ^68^Ga-satoreotide trizoxetan imaging. There was only one reading where readers diverged on scoring; one reader preferred one image because of higher lesion conspicuity, and the other reader preferred the alternative image because of the ability to identify more lesions.

**Conclusions:**

Binary visual reading, which was associated with a low inter-reader variability, has further supported that the optimal administered radioactivity of ^68^Ga-satoreotide trizoxetan was 100–200 MBq with a peptide mass up to 50 µg.

*Trial registration* ClinicalTrials.gov, NCT03220217. Registered 18 July 2017, https://clinicaltrials.gov/ct2/show/NCT03220217

## Background

The use of ^68^Ga-radiolabelled somatostatin receptor (SSTR) agonists, such as ^68^Ga-DOTATATE and ^68^Ga-DOTATOC, for the positron emission tomography/computed tomography (PET/CT) imaging of neuroendocrine tumours (NETs) is well-established in clinical practice, not only for the localisation and staging of NETs, but also as a theranostic tool or biomarker for assessing the potential response to peptide receptor radionuclide therapy [1]. In the evaluation of new imaging techniques and novel tracers, image quality is usually assessed using a 5-point Likert scale: the exact methodology of which is unique to each clinical trial, but standardly “1” is poor quality and “5” is high quality [2–9]. Despite its widespread use, the Likert scale in biomedical imaging assessment is limited by inter-reader variability, fixed upper and lower limits, and a lack of mathematical and statistical validity [10–12].

To the authors’ knowledge, the binary evaluation of image quality in nuclear medicine (wherein an individual reader sees two images simultaneously and designates them as having either optimal or non-optimal image quality) has not been previously described, although binary reading of PET images has been used in the evaluation of the presence or absence of amyloid in Alzheimer's disease [13, 14]. Unlike the Likert assessment, an important advantage of binary visual reading is its simplicity and convenience, as it removes the need for images to be reviewed at different times, particularly in studies with a small number of images. These features suggest that binary contemporaneous visual reading might be used to optimise clinical development of PET/CT imaging agents.

The use of radiolabelled SSTR antagonists rather than agonists has the potential to improve the PET/CT imaging of NETs, because SSTR antagonists bind to significantly more receptor sites than SSTR agonists [15]. ^68^Ga-satoreotide trizoxetan (also known as ^68^Ga-IPN01070, ^68^Ga-NODAGA-JR11, or ^68^Ga-OPS202) is a novel SSTR antagonist recently evaluated in a prospective phase I/II imaging study conducted in 12 patients with well-differentiated gastroenteropancreatic (GEP) NETs. The authors showed that compared with ^68^Ga-DOTATOC, ^68^Ga-satoreotide trizoxetan exhibits substantially higher tumour-to-background ratios and sensitivity for detecting liver metastases [16, 17]. Subsequently, a phase II study was conducted to confirm the optimal administered peptide mass and radioactivity of ^68^Ga-satoreotide trizoxetan in patients with GEP-NETs [18].

The primary results of this phase II study [18] showed that the ratio of the number of lesions detected by ^68^Ga-satoreotide trizoxetan imaging to the number of lesions detected by contrast-enhanced CT was overall consistent across different peptide mass and radioactivity range combinations (5–20 or 30–45 µg with one of three gallium-68 radioactivity ranges: 40–80, 100–140, or 160–200 MBq). However, a trend towards a lower ratio in the liver was noted for the radioactivity range of 40–80 MBq compared to higher radioactivity ranges. There were no safety concerns associated with ^68^Ga-satoreotide trizoxetan.

When comparing different peptide mass and radioactivity combinations of an imaging agent, a key aspect is the evaluation of image quality. We hypothesised that a binary visual reading technique comparing the images contemporaneously (rather than a multi-scoring technique in multiple review sessions) could reduce the readers’ workload and inter-reader variability, and thus improve the key endpoint assessment. Accordingly, here, we report the binary visual reading results in the aforementioned phase II study aimed to determine the optimal peptide mass and radioactivity ranges for ^68^Ga-satoreotide trizoxetan in patients with GEP-NETs.

## Methods

### Study design and patient population

**Fig. 1:**
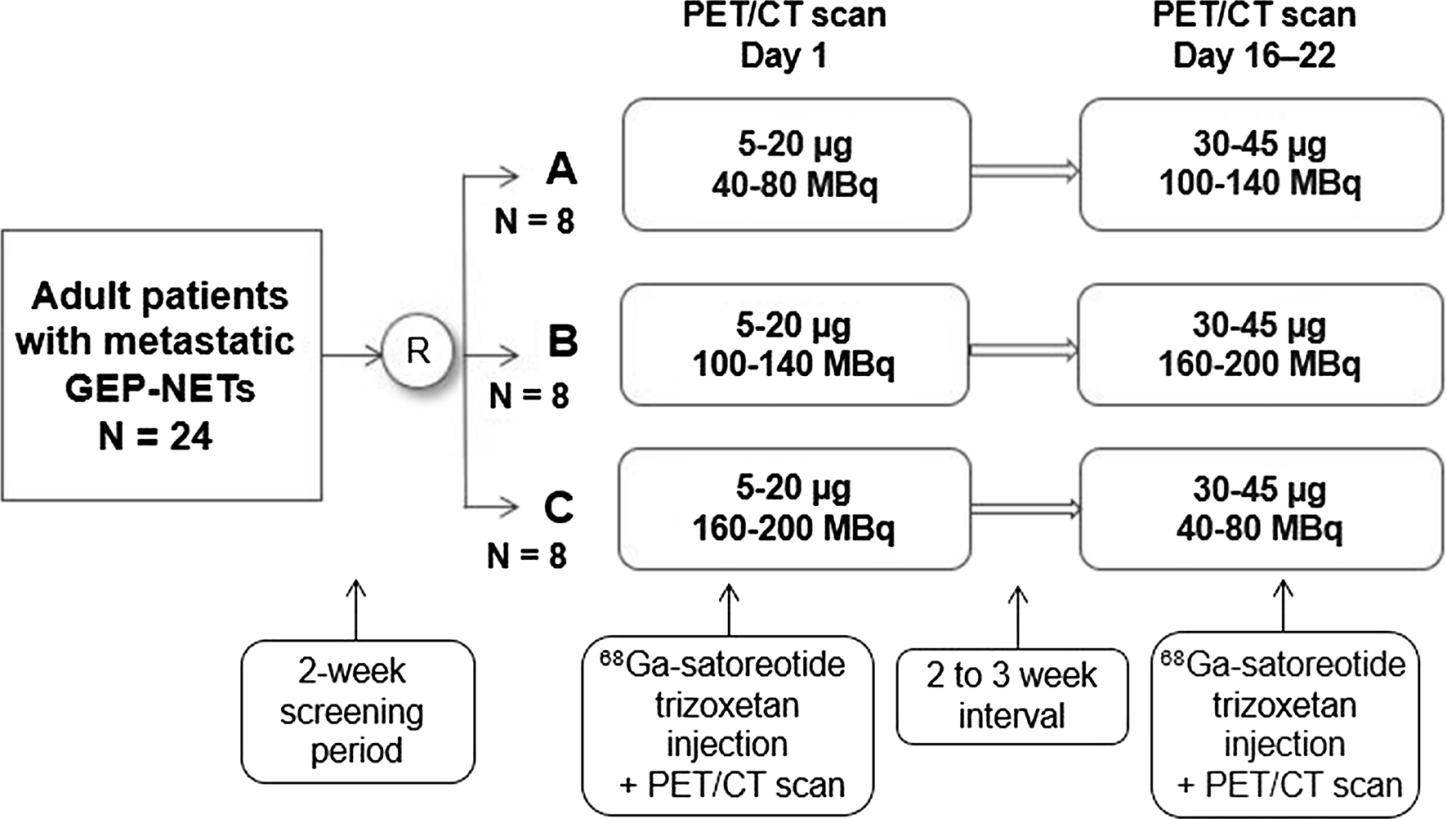
Study design. Abbreviations: CT, computed tomography; GEP, gastroenteropancreatic; NET, neuroendocrine tumour; PET, positron emission tomography; R, randomised

This prospective, multinational, multicentre, dose-confirmation phase II study (ClinicalTrials.gov identifier: NCT03220217; EudraCT No.: 2016-004928-39) was conducted between September 2017 and August 2019, using an open-label, reader-blinded, 2 × 3 factorial design. The methodology and primary results have been reported elsewhere [18]. In summary, a total of 24 adult patients with well-differentiated, metastatic, grade 1/2, SSTR-positive GEP-NETs were randomised (1:1:1) to one of three arms (8 patients per arm) with six peptide mass/radioactivity range combinations of ^68^Ga-satoreotide trizoxetan. Essentially, all patients received two different doses of ^68^Ga-satoreotide trizoxetan in one of the three arms on two consecutive visits separated by a 2–3-week interval, thereby providing a pair of PET/CT images for each patient (Fig. 1). The administered peptide mass range for all three arms was 5–20 µg on visit 1 (day 1 of the study) and 30–45 µg on visit 2 (day 16–22). The radioactivity range was for:

- Arm A: 40–80 MBq (visit 1) and 100–140 MBq (visit 2)
- Arm B: 100–140 MBq (visit 1) and 160–200 MBq (visit 2)
- Arm C: 160–200 MBq (visit 1) and 40–80 MBq (visit 2)

All patients had a SSTR agonist PET/CT scan acquired within the previous six months of study enrolment. The main exclusion criteria were the presence of < 5 lesions in total and > 25 lesions per organ detected by the screening SSTR PET/CT scan in either the liver, lymph nodes, bones, or lungs; treatment with a somatostatin analogue within 28 days before ^68^Ga-satoreotide trizoxetan administration; and any condition that might preclude the proper performance of a PET and/or CT scan (e.g., patient body weight > 110 kg; inability to raise arms for prolonged imaging purposes or to lie still for the entire imaging time).

The study was performed in four investigational sites in Austria, Denmark and the USA, and was conducted in accordance with the Declaration of Helsinki as well as the Good Clinical Practice (GCP) guidelines. Ethics committee approval was obtained at each participating site, and all patients provided written informed consent.

### PET/CT imaging protocol

An image core laboratory (Rad-MD, New York City, New York, USA) provided a comprehensive, manufacturer-specific image acquisition manual, which detailed the requirements for the investigator site personnel to ensure inter-site consistency of image acquisition protocols across scanners and sites.

On both day 1 and day 16–22 study visits, whole-body PET/CT imaging (from skull base to mid-thigh) was performed for all patients 50–70 min after the intravenous injection of ^68^Ga-satoreotide trizoxetan, using either Siemens Biograph dedicated PET/CT scanners or GE Discovery 690 PET/CT scanners at each of the four study centres. Three-dimensional PET scans were acquired in list mode, with a 4 min per bed position and a 5-slice overlap. CT scan was performed using intravenous iodinated contrast media, with a maximum slice thickness of 3 mm.

The effective administered activity of ^68^Ga-satoreotide trizoxetan (defined as the activity in the syringe before injection minus the residual activity in the empty syringe after injection) was used for PET image reconstruction. PET/CT data were volume rendered with maximum intensity projection for PET and direct volume rendering for CT. No fasting or dietary restrictions were imposed on study participants prior to PET/CT imaging.

All trial sites qualified their PET scanners using the Clinical Trials Network (CTN) of the Society of Nuclear Medicine and Molecular Imaging (SNMMI) Scanner Validation Program to ensure baseline common quality control metrics for PET scanners used across the sites [19]. Before imaging patients at each study site, the PET/CT scanner was cross-calibrated to a well counter calibrated for gallium-68using a homogeneously filled phantom; the phantom was used to ensure that PET/CT scanner images were comparable and reproducible across the study sites for image noise and texture. PET imaging data were collected and confirmed by the SNMMI CTN, to ensure the standardised uptake values (SUV) used in the trial were reliable.

In addition, quality control by the imaging core laboratory was performed on all acquired ^68^Ga-satoreotide trizoxetan PET/CT scans. In case of detection of patient movement-related artifacts, a scan repeat would be requested, or a patient would be replaced.

### Qualitative imaging analysis

Following anonymisation, ^68^Ga-satoreotide trizoxetan PET/CT scans were all sent to an imaging core laboratory (Keosys, Nantes, France), and evaluated centrally. A total of 48 PET/CT images were read: two for each of the 24 patients acquired at the two study visits, with 8 images in each peptide mass/radioactivity range combination. Prior to initiating the PET/CT image reads, a formal study training day, including a “calibration” agreement session, was conducted in New York, with all readers present. ^68^Ga-satoreotide trizoxetan images acquired in the Nicolas et al. (2018) phase I/II study of 12 patients with well-differentiated GEP-NETs [16, 17] were used for training, in a blinded manner by all readers to evaluate inter-reader consistency and to reach consensus on areas of interpretative difference.

**Fig. 2:**
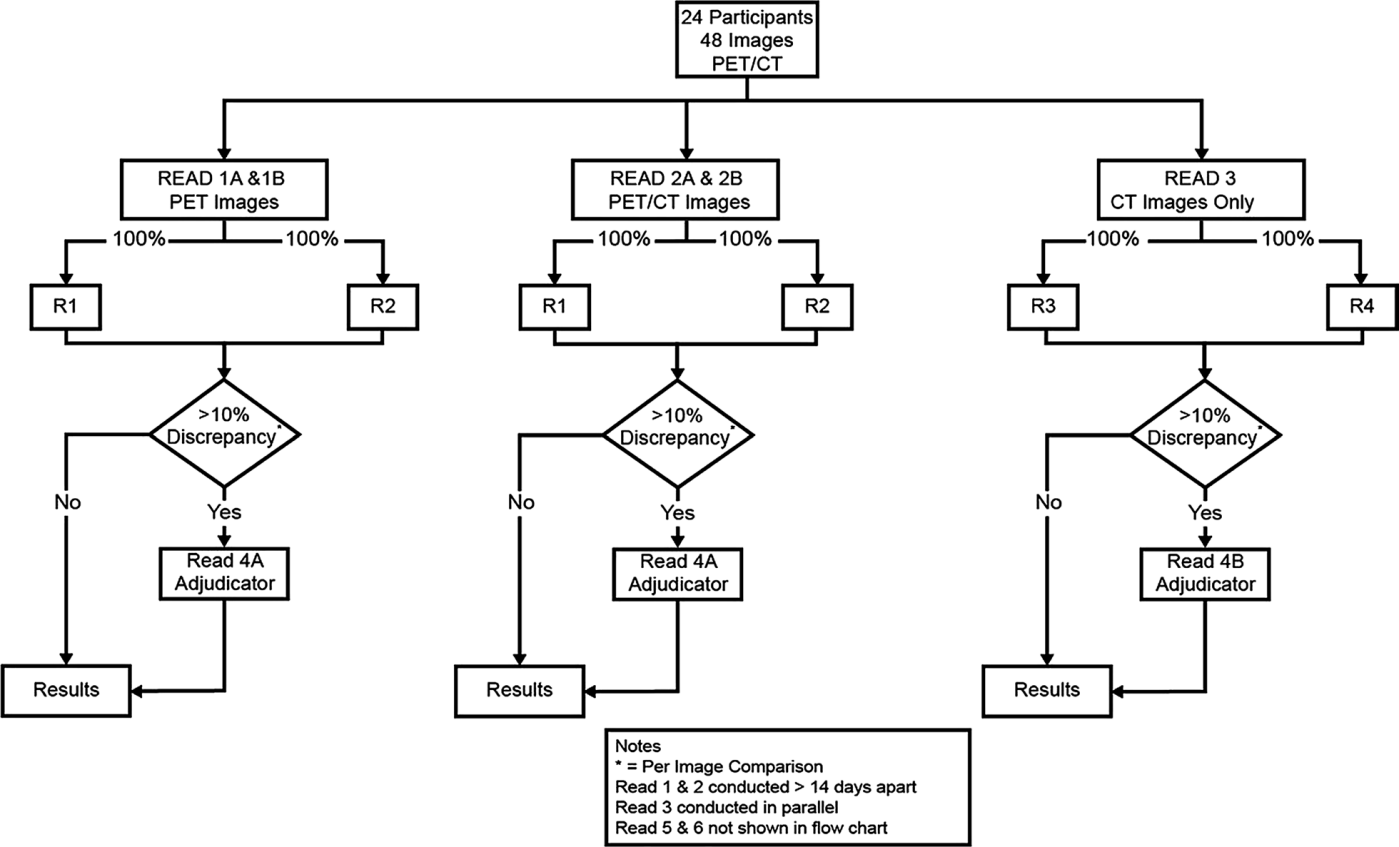
Read design. Abbreviations: CT, computed tomography; PET, positron emission tomography; R, reader

The read design schema is shown in Fig. 2, with a total of six read sessions. All readers were blinded to all patient identification, clinical data, and to any information related to the study site, visit, and administered peptide mass and radioactivity. To maintain consistency, the patients’ PET/CT scans were read in batches of approximately six patients. The ^68^Ga-satoreotide trizoxetan PET images were read either alone (read 1A and 1B) or as a fused PET/CT scan (read 2A and 2B) by two different pairs of nuclear medicine physicians. In parallel, the contrast-enhanced CT scans, which were used for statistical comparisons, were independently read by two radiologists (read 3). The ^68^Ga-satoreotide trizoxetan PET and PET/CT images were randomised in presentation sequence (visit 1 or visit 2) so as to blind the readers from the timepoint and administered dose, with a minimum interval of 14 days between the two reads in an attempt to decrease reader image memory and possible recall bias. The purpose of the read 1A, read 2A, and read 3 sessions was to count the number of lesions per organ on each patient scan, with the use of an adjudication paradigm during read session 4 in case of a > 10% discrepancy between the two independent readers in the lesion count.

Reads 1B and 2B were separate image quality read sessions where the two ^68^Ga-satoreotide trizoxetan scans acquired from the same patient were presented to the two experienced nuclear medicine physicians for visual assessment, using a split screen technique. Both physicians read the images independently without reference to the other. Each reader directly compared the paired patient images contemporaneously, recording which of the pair provided optimal image quality. Both readers had the ability to use window/level manipulation on each reviewed image. ^68^Ga-satoreotide trizoxetan PET and PET/CT scans were scored in two different sessions using a binary system (0 for non-optimal image quality and 1 for optimal image quality). For each patient pair of ^68^Ga-satoreotide trizoxetan scans, one or both images could score 1. Both primary readers’ scores were subsequently combined providing a maximum total image quality score (IQS) of 16 per peptide mass/radioactivity range combination (8 images × 2 readers). No adjudications were held for the image quality reads (reads 1B and 2B).

### Quantitative analysis

During a subsequent reading session (read 5), another independent, qualified reader, not involved in the ^68^Ga-satoreotide trizoxetan or contrast-enhanced CT image reads, reviewed all 48 PET/CT scans, in order to perform the semi-quantitative measurements of maximum standardised uptake values (SUV_max_) to support the qualitative imaging analysis. Spherical regions of interest were manually drawn by the reader in up to the five most avid lesions detected in the primary tumour site, liver, lymph nodes, bones, and lungs. As a reference tissue, the SUV_mean_ of non-tumoural liver parenchyma was measured in a 3-cm region of interest in all ^68^Ga-satoreotide trizoxetan PET/CT scans while avoiding large vessels. The tumour-to-liver ratio was calculated as lesion SUV_max_/non-tumoural liver parenchyma SUV_mean_ for each pre-defined anatomic region, and was determined across the different peptide mass and radioactivity ranges.

### Statistical analysis

This was a descriptive analysis. As prespecified in the Statistical Analysis Plan, 24 patients were to be included in the per-protocol population (the first eight patients in each arm) to ensure balanced arms and paired reading of images for comparability across doses. Categorical variables were expressed as counts and percentages, and continuous variables as mean ± standard deviation or median (range), depending on the distribution of values. All imaging endpoints were also evaluated based on the administered radioactivity (MBq) per patient’s baseline body weight (kg) in four distinct subgroups: 0.69–0.97, 0.97–1.55, 1.55–2.09, and 2.09–3.72 MBq/kg.

Statistical analyses were performed using SAS version 9.4 (SAS Institute Inc, Cary, NC). Missing values were not replaced.

## Results

### Patients

The baseline demographic characteristics of the study population are presented in Table 1. Overall, 21/24 (87.5%) patients had intestinal NETs, and 3 (12.5%) pancreatic NETs. The liver (in 22/24 patients; 91.7%) and lymph nodes (19/24; 79.2%) were the most frequent locations of metastases.

**Table 1:** Baseline demographic characteristics in the per-protocol population

Parameter	Arm A (*N* = 8)	Arm B (*N* = 8)	Arm C (*N* = 8)	Overall (*N* = 24)
Age, years	71.5 (54–84)	65.5 (60–72)	60.5 (48–76)	62.5 (48–84)
Sex				
Male	6 (75.0)	3 (37.5)	7 (87.5)	16 (66.7)
Female	2 (25.0)	5 (62.5)	1 (12.5)	8 (33.3)
Body weight, kg	81.0 (77–98)	87.5 (52–109)	87.5 (60–105)	85.0 (52–109)
Height, cm	175.5 (162–189)	171.0 (159–185)	177.5 (171–189)	176.0 (159–189)
BMI, kg/m^2^	26.4 (24–35)	28.8 (21–34)	26.6 (19–31)	26.7 (19–35)
ECOG performance status				
0 (normal activity)	7 (87.5)	7 (87.5)	7 (87.5)	21 (87.5)
1 (restricted activity)	1 (12.5)	1 (12.5)	1 (12.5)	3 (12.5)

Data are presented as *n* (%) or median (range). Percentages are calculated as n/N

*BMI* body mass index, *ECOG* Eastern Cooperative Oncology Group

All 24 patients had a prior SSTR scan acquired within a median of 1.6 months (range, 0.1–6.0 months) from screening: 14 (58.3%) with ^68^Ga-DOTATOC, 9 (37.5%) with ^64^Cu-DOTATATE, and 1 (4.2%) with ^68^Ga-DOTATATE. The total number of SSTR-positive lesions, expressed as median (range), detected by prior SSTR agonist scans was 14.5 (6.0–94.0), with 1.0 lesions (0–1.0) in the primary tumour site, 9.5 (0–37.0) in the liver, 5.0 (0–36.0) in the lymph nodes, and 0.5 (0–38.0) in the bones. Three patients were enrolled with over 30 lesions identified in the liver or in the lymph nodes.

### Image quality

The IQSs are presented in Table 2 for both the ^68^Ga-satoreotide trizoxetan PET/CT and PET only reads. The IQSs were overall similar across the three radioactivity ranges for the PET/CT reads. However, for the PET only reads, the total IQS was lower in the 40–80 MBq radioactivity range (56.3%) compared to the 100–140 MBq (90.6%) and 160–200 MBq (81.3%) ranges. With regard to the evaluated peptide mass ranges, the total IQSs were 60.4% in the 5–20 µg peptide mass range and 83.3% in the 30–45 µg peptide mass range for the PET/CT readings. For the PET only readings, the total IQSs were 70.8% and 81.3% in the 5–20 and 30–45 µg ranges, respectively.

**Table 2:** Image quality scores for ^68^Ga-satoreotide trizoxetan PET/CT and PET, by peptide mass and radioactivity range

Peptide mass range	Radioactivity range			
	40–80 MBq	100–140 MBq	160–200 MBq	Total
PET/CT readings				
5–20 µg	9/16 (56.3)	10/16 (62.5)	10/16 (62.5)	29/48 (60.4)
30–45 µg	13/16 (81.2)	14/16 (87.5)	13/16 (81.3)	40/48 (83.3)
Total	22/32 (68.8)	24/32 (75.0)	23/32 (71.9)	–
PET readings				
5–20 µg	7/16 (43.8)	14/16 (87.5)	13/16 (81.3)	34/48 (70.8)
30–45 µg	11/16 (68.8)	15/16 (93.8)	13/16 (81.3)	39/48 (81.3)
Total	18/32 (56.3)	29/32 (90.6)	26/32 (81.3)	–

During the image quality readings, each image was assigned a “1” (for optimal image quality) or a “0” (for non-optimal image quality). For each pair of patient images of ^68^Ga-satoreotide trizoxetan obtained on day 1 and day 16–22 of the study, one or both images could score “1”. The numerator of the image quality score is the sum of both readers’ scores, and the denominator is the total number of PET/CT and PET scans that were read and scored

Data are presented as *n*/*N* (%)

*CT* computed tomography, *PET* positron emission tomography

IQSs based on the administered radioactivity per patient’s body weight are presented in Table 3. While the overall results were similar for the evaluated quartiles of body mass, the IQSs in the 5–20 µg range were in general lower than that in the 30–45 µg peptide mass range, particularly for the ^68^Ga-satoreotide trizoxetan PET/CT readings.

**Table 3:** Image quality scores for ^68^Ga-satoreotide trizoxetan PET/CT and PET, by radioactivity per patient’s body weight

	0.69–0.97 MBq/kg	0.97–1.55 MBq/kg	1.55–2.09 MBq/kg	2.09–3.72 MBq/kg	Total
PET/CT readings					
5–20 µg	9/16 (56.3)	6/10 (60.0)	7/12 (58.3)	7/10 (70.0)	29/48 (60.4)
30–45 µg	9/12 (75.0)	9/10 (90.0)	11/14 (78.6)	11/12 (91.7)	40/48 (83.3)
Total	18/28 (64.3)	15/20 (75.0)	18/26 (69.2)	18/22 (81.8)	–
PET readings					
5–20 µg	7/16 (43.8)	9/10 (90.0)	9/12 (75.0)	9/10 (90.0)	34/48 (70.8)
30–45 µg	8/12 (66.7)	8/10 (80.0)	12/14 (85.7)	11/12 (91.7)	39/48 (81.3)
Total	15/28 (53.6)	17/20 (85.0)	21/26 (80.8)	20/22 (90.9)	–

During the image quality readings, each image was assigned a “1” (for optimal image quality) or a “0” (for non-optimal image quality). The numerator of the image quality score is the sum of both readers’ scores, and the denominator is the total number of PET/CT and PET scans that were read and scored

Data are presented as *n*/*N* (%)

*CT* computed tomography, *PET* positron emission tomography

During the image quality read sessions, there was only one reading where the readers diverged on scoring; one reader preferred one image because of higher lesion conspicuity, and the other reader preferred the alternative image because of the ability to identify more lesions. All other reads were congruent in that the readers agreed on at least one of the images having an IQS of 1. Compared to ^68^Ga-satoreotide trizoxetan PET/CT readings, both primary readers reported that it was overall more difficult to localise hepatic and pulmonary lesions in PET only readings.

**Fig. 3:**
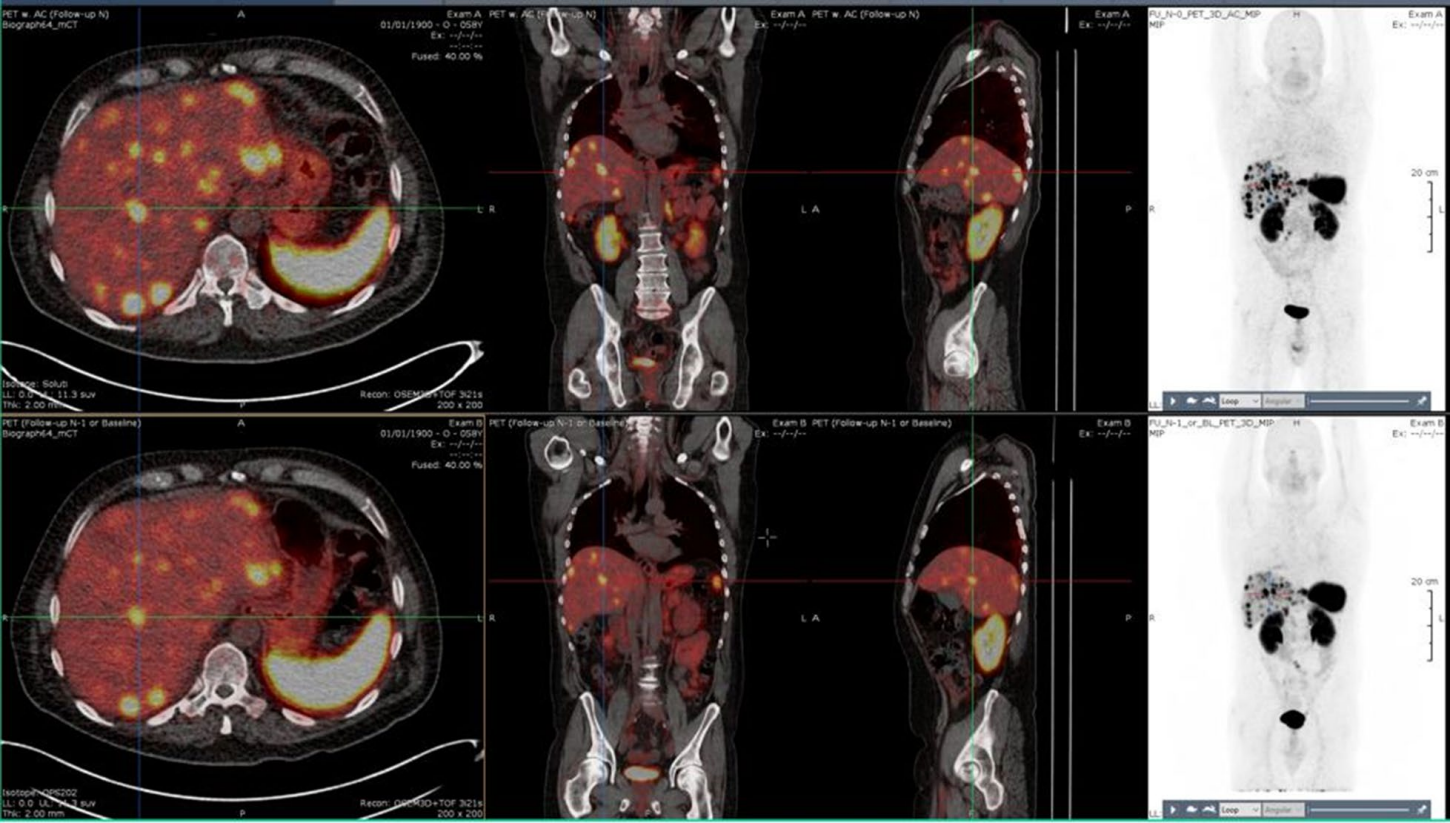
Two ^68^Ga-satoreotide trizoxetan PET/CT fused images with maximum intensity projection of the same patient acquired at two different timepoints separated by 2–3 weeks. In the top panel, the patient had received ^68^Ga-satoreotide trizoxetan at 16 µg/165 MBq. In the lower panel, the patient had received ^68^Ga-satoreotide trizoxetan at 32 µg/72 MBq. Abbreviations: CT, computed tomography; PET, positron emission tomography

A pair of ^68^Ga-satoreotide trizoxetan PET/CT fused images acquired from the same patient at the two study timepoints is illustrated in Fig. 3.

### Semi-quantitative assessment

SUV_max_ and the tumour-to-liver ratio (SUV_max_/SUV_mean_) were evaluated on all ^68^Ga-satoreotide trizoxetan scans, and the results were compared across the peptide mass and radioactivity range combinations, with no consistent pattern in the median values identified (Table 4). Similarly, when assessing tumour-to-liver ratio by peptide mass range and radioactivity range (Table 5), no notable differences in the mean and median ratios were observed. There was also no apparent relationship between the radioactivity range per patient’s body weight and SUV_max_ of the liver and lymph nodes (Table 6).

**Table 4:** SUV_max_ and tumour-to-liver ratio for the liver and lymph nodes, by combination

		Arm A (*N* = 8)		Arm B (*N* = 8)		Arm C (*N* = 8)	
Organ	Statistic	5–20 µg 40–80 MBq	30–45 µg 100–140 MBq	5–20 µg 100–140 MBq	30–45 µg 160–200 MBq	5–20 µg 160–200 MBq	30–45 µg 40–80 MBq
SUV_max_							
Liver	*n*	5	5	5	5	8	8
	Median (range)	24.2 (18.25–49.16)	22.9 (18.01–59.83)	9.5 (6.74–63.68)	16.0 (9.56–78.43)	12.4 (6.95–30.07)	17.7 (10.62–30.28)
Lymph nodes	n	3	3	6	6	5	5
	Median (range)	24.7 (19.52–40.74)	35.7 (16.69–41.11)	28.5 (9.03–83.06)	27.7 (5.25–53.79)	13.8 (6.08–21.73)	12.7 (6.15–21.33)
Tumour-to-liver ratio (SUV_max_/SUV_mean_)							
Liver	*n*	5	5	5	5	8	8
	Median (range)	9.9 (8.51–37.00)	8.4 (7.27–28.31)	6.0 (4.85–40.14)	7.0 (5.08–48.79)	7.2 (3.28–17.98)	9.3 (5.60–32.37)
Lymph nodes	n	3	3	6	6	5	5
	Median (range)	18.6 (6.17–27.43)	16.9 (4.83–21.50)	9.9 (4.42–26.46)	13.6 (2.26–25.14)	9.8 (5.30–20.45)	8.2 (5.56–28.98)

*n* corresponds to the number of patients with lesions in either the liver or the lymph nodes

The number of patients with lesions in the bones, lungs, and the primary tumour site was too small (≤ 3 in each category) to allow a meaningful interpretation of PET/CT scans for these organs

*CT* computed tomography, *PET* positron emission tomography, *SUV* standardised uptake value

**Table 5:** Tumour-to-liver (SUV_max_/SUV_mean_), by peptide mass and radioactivity range

		Peptide mass range		Radioactivity range		
Organ	Statistic	5–20 µg (*N* = 24)	30–45 µg (*N* = 24)	40–80 MBq (*N* = 16)	100–140 MBq (*N* = 16)	160–200 MBq (*N* = 16)
Primary tumour site	*n*	6	5	3	5	3
	Mean ± SD	32.1 ± 39.22	29.2 ± 28.01	41.4 ± 53.08	30.1 ± 27.67	21.3 ± 27.78
	Median (range)	12.6 (4.94–102.40)	16.5 (5.57–64.98)	16.2 (5.67–102.40)	16.5 (5.31–64.98)	5.6 (4.94–53.36)
Liver	*n*	18	18	13	10	13
	Mean ± SD	11.5 ± 10.51	13.6 ± 11.72	12.9 ± 9.94	13.0 ± 11.87	11.8 ± 12.20
	Median (range)	8.6 (3.28–40.14)	8.4 (5.08–48.79)	9.9 (5.60–37.00)	7.7 (4.85–40.14)	7.0 (3.28–48.79)
Lymph nodes	*n*	14	14	8	9	11
	Mean ± SD	13.2 ± 8.52	13.1 ± 8.98	13.8 ± 9.78	13.7 ± 8.93	12.3 ± 8.18
	Median (range)	10.1 (4.42–27.43)	8.6 (2.26–28.98)	8.6 (5.56–28.98)	10.8 (4.42–26.46)	9.8 (2.26–25.14)

*n* corresponds to the number of patients with lesions in either the primary tumour site, liver, or the lymph nodes

The number of patients with lesions in the bones and lungs was too small (≤ 1 in each category) to allow a meaningful interpretation of PET/CT scans for these organs

*CT* computed tomography, *PET* positron emission tomography, *SD* standard deviation, *SUV* standardised uptake value

**Table 6:** SUV_max_ of the liver and lymph nodes, by radioactivity per patient’s body weight

Organ	Statistic	0.69–0.97 MBq/kg	0.97–1.55 MBq/kg	1.55–2.09 MBq/kg	2.09–3.72 MBq/kg
Liver	*N*	11	6	9	8
	Mean ± SD	24.4 ± 9.46	13.8 ± 5.49	16.1 ± 7.25	23.3 ± 17.77
	Median (range)	23.8 (15.0–49.0)	12.6 (8.0–22.0)	14.8 (7.0–26.0)	14.1 (10.0–60.0)
Lymph nodes	*N*	5	6	7	8
	Mean ± SD	25.5 ± 8.72	34.3 ± 26.46	29.2 ± 15.75	17.9 ± 11.81
	Median (range)	21.3 (20.0–41.0)	28.5 (12.0–83.0)	21.7 (13.0–54.0)	13.0 (5.0–36.0)

*n* corresponds to the number of patients with lesions in either the liver or the lymph nodes

The number of patients with lesions in the primary tumour site and bones was too small (≤ 3 in each category) to allow a meaningful interpretation of PET/CT scans for these organs

*CT* computed tomography, *PET* positron emission tomography, *SD* standard deviation, *SUV*_*max*_ maximum standardised uptake value

## Discussion

In this study, we evaluated a binary visual reading methodology to reduce the readers’ workload and inter-reader variability, and improve study endpoints’ assessment following administration of a ^68^Ga-labelled radiopharmaceutical. The main finding was that a binary scoring system evaluating image quality in a contemporaneous manner was indeed useful in appraising image quality in a reader-centric manner with a low inter-reader variability, and can consequently be recommended for future evaluation of imaging studies.

The evaluation of image quality in product development and clinical trials is most commonly assessed by using the 5-point Likert scale as a qualitative index [20–22] requiring multiple reader sessions. A Likert scoring methodology, while providing the appearance of a robust evaluation, has several practical and methodological challenges. Most importantly, it requires user (reader) calibration to define the exact metric to each unit of score. Without careful definition and calibration, there will be mismatch errors among the readers which might lead to significant statistical noise, as there is potentially more discrepancy in the interpretation of the score values than within the images themselves. Furthermore, the use of a Likert scale requires a relatively large sample size. By comparison, the methodology presented here relies on a small pre-determined balanced approach requiring far fewer patients to be enrolled and a forced direct image comparison creating an IQS. The approach of comparing two doses of ^68^Ga-satoreotide trizoxetan using a split screen technique simplified the readers’ work and provided more robust responses to the challenge of selecting one of two images. The binary visual reading technique, while described elsewhere, does not, surprisingly, appear to have been widely adopted in the imaging field and in the nuclear medicine arena. The obvious advantage of a reduced number of patients being involved makes a compelling argument for future consideration of binary contemporaneous visual reading for image evaluation. As noted, there was only one discrepant reading between the two primary readers in our study, supporting the hypothesis that a binary scoring system would lead to low inter-reader variability and evaluate image quality in a reader-centric manner.

Besides the adoption of a binary contemporaneous visual reading scoring system, a blinded independent central review, which has been reported to increase the reproducibility of study results [20, 23], was performed to optimise qualitative image evaluation of ^68^Ga-satoreotide trizoxetan PET/CT scans in this study. In addition, by omitting clinical and dosing data as well as information on the study site and timepoints, the blinded reads provided pure imaging data results. Reading/interpretation bias was further reduced by the adoption of a multiple randomisation scheme. Of note, when evaluating novel radiopharmaceuticals, it is important that the read methodology is carefully described to meet the needs of regulatory agencies such as the United States Food and Drug Administration [24–27].

A key contributing factor to ensuring successful image quality assessment is carefully designed acquisition protocols, with strict quality control. This was accomplished with the support of the imaging core labs (Rad-MD, Keosys, and SNMMI CTN) and the investigator sites ensuring that patients were imaged according to the study protocol and GCP guidelines. Another key aspect is the reader training/calibration, which was conducted face to face, and included the use of images acquired in the aforementioned Nicolas et al. (2018) phase I/II study [16, 17] to accustom the readers to evaluating ^68^Ga-satoreotide trizoxetan PET/CT and to provide initial inter-reader variability information.

In terms of radioactivity ranges, the IQSs for the ^68^Ga-satoreotide trizoxetan PET/CT and PET only scans were lower for the 40–80 MBq range, especially with the PET only images. These results, along with the other published data from the study [18], confirmed that this radioactivity range of ^68^Ga-satoreotide trizoxetan cannot be recommended for phase III development, particularly given that it has never been evaluated in prior studies with other ^68^Ga-labelled agents. The combination of 40–80 MBq and 5–20 µg appears to have the poorest quality of reads, as illustrated by the IQSs for ^68^Ga-satoreotide trizoxetan PET/CT and PET only scans when compared to other peptide mass and radioactivity range combinations, suggesting a potential peptide mass dose effect. However, once the combination of 40–80 MBq and 5–20 µg is ignored, the differences in IQSs across the evaluated peptide mass and radioactivity range combinations are reduced, particularly for the PET only reads. Moreover, the semi-quantitative analysis showed no notable differences in the mean and median values of SUV_max_ and SUV_max_/SUV_mean_ for the liver and lymph nodes across the evaluated peptide mass and radioactivity range combinations. Therefore, the overall results lead to the conclusion that peptide mass does not influence ^68^Ga-satoreotide trizoxetan imaging. This is in line with the Nicolas et al. (2018) phase I/II imaging study [16, 17] in which two peptide masses (15 and 50 µg) of ^68^Ga-satoreotide trizoxetan were injected in 12 patients with GEP-NETs. There were no significant differences between the two peptide masses in the numbers of malignant liver or lymph node lesions detected per patient and in tumour uptake [16, 17].

Data regarding the optimal peptide mass range of radiolabelled SSTR antagonists used for functional NET imaging are scarce [28, 29]. Currently, a peptide mass up to 50 µg is used for imaging, as per current administration guidelines for ^68^Ga-labelled radiopharmaceuticals [30], to avoid the risk of receptor saturation and to allow accurate quantification of the receptor density [28, 29]. However, this recommended peptide mass range is more reflective of common practice than of evidence from controlled studies [28]. Nevertheless, the results of the present study support an optimal peptide mass of ^68^Ga-satoreotide trizoxetan up to 50 µg for the diagnostic imaging of GEP-NETs.

This present study also supports the administration of a radioactivity range of ^68^Ga-satoreotide trizoxetan between 100 and 200 MBq, which is in line with the Oncology Committee of the European Association of Nuclear Medicine’s procedural guidelines for PET/CT tumour imaging with ^68^Ga-conjugated peptides [30]. To ensure a high diagnostic quality of ^68^Ga-satoreotide trizoxetan PET/CT examination and to avoid negative impacts on image quality or lesion detectability [5], it is not recommended to reduce the administrated radioactivity below 100 MBq. Of note, in a prospective study among 24 patients with SSTR-positive NETs who received a single intravenous injection of ^68^Ga-DOTATOC administered at a mean dose of 120 MBq, both ^68^Ga-DOTATOC PET/CT and PET/magnetic resonance imaging were associated with good image quality and detectability of focal PET lesions on both a patient basis and organ system basis [31]. In the present study, the total IQS for ^68^Ga-satoreotide trizoxetan PET scans was higher in the 100–140 MBq radioactivity range (90.6%) compared to the 160–200 MBq (81.3%) range, suggesting a possibility of narrowing the radioactivity window of ^68^Ga-satoreotide trizoxetan from 100–200 to 100–140 MBq. However, adopting the wider, guideline-recommended radioactivity range of 100–200 MBq offers increased flexibility and feasibility in routine clinical practice, while maintaining similarity in dosing to other gallium-68-labelled products [30]. The absence of a clear radioactive dose–response relationship in the present study might be related to factors such as heterogeneity in receptor density, hypoxia, interstitial pressure, necrosis, and tumour heterogeneity [32].

This study was mainly limited by a small sample size, the use of a descriptive statistical analysis only, the lack of a direct comparison between binary contemporaneous visual reading and the Likert scale system, and the absence of a comparator arm to evaluate the repeatability of the imaging results. In addition, inter- and intra-reader variabilities were not quantified. However, to minimise inter-reader variability, a full reader training which included the binary visual reading of ^68^Ga-satoreotide trizoxetan PET/CT scans acquired in the Nicolas et al. (2018) phase I/II study [16, 17] was undertaken prior to the start of the study. Analyses of tumour SUV were also restricted to measurement of SUV_max_. Although semi-quantitative assessments using SUV_max_ may allow a more uniform evaluation of the diagnostic value of ^68^Ga-satoreotide trizoxetan [33], the number of patients in the present study with lesions in the bones, lungs, and the primary tumour site was too small to draw conclusions for these organs on the differences in the mean and median values of SUV_max_ and SUV_max_/SUV_mean_ across the administered peptide mass and radioactivity ranges. This study nevertheless had several strengths, particularly a robust research design allowing inter- and intra-individual comparisons of different peptide mass and radioactivity range combinations, the adoption of a novel read methodology providing direct image comparisons and consequently removing the subjectivity of inter-timepoint variations, the utilisation of a blinded independent central review, the inclusion of both quantitative and qualitative imaging measures, and a uniform PET/CT imaging protocol across all study sites. Based on the results of the present study, ^68^Ga-satoreotide trizoxetan administered at 100–200 MBq with a peptide mass up to 50 μg will be further evaluated in larger prospective clinical studies.

## Conclusions

This study provided the opportunity to develop a simple and precise image quality scoring system, associated with a low inter-reader variability. This binary contemporaneous scoring system can consequently be recommended for future evaluation of imaging studies. The read quality results in the present study, along with the results reported elsewhere [18], confirm an optimal administered radioactivity of ^68^Ga-satoreotide trizoxetan of 100–200 MBq with a peptide mass up to 50 μg for future clinical development.

## Data Availability

Where patient data can be anonymised, Ipsen will share all individual participant data that underlie the results reported in this article with qualified researchers who provide a valid research question. Study documents, such as the study protocol and clinical study report, are not always available. Proposals should be submitted to DataSharing@Ipsen.com, and will be assessed by a scientific review board. Data are available beginning 6 months and ending 5 years after publication; after this time, only raw data may be available.

## Abbreviations

CT: computed tomography
CTN: clinical trials network
GCP: good clinical practice
GEP: gastroenteropancreatic
IQS: image quality score
NET: neuroendocrine tumour
PET: positron emission tomography
SNMMI: society of nuclear medicine and molecular imaging
SSTR: somatostatin receptor
SUV: standardised uptake value
SUV_max_: maximum standardised uptake value
